# Baseline hospital performance and the impact of medical emergency teams: Modelling vs. conventional subgroup analysis

**DOI:** 10.1186/1745-6215-10-117

**Published:** 2009-12-19

**Authors:** Jack Chen, Arthas Flabouris, Rinaldo Bellomo, Ken Hillman, Simon Finfer, MERIT investigators for the Simpson Centre and the ANZICS Clinical Trial Group

**Affiliations:** 1 The Simpson Centre for Health Services Research, University of New South Wales, Sydney, New South Wales, Australia; 2 Intensive Care Unit, Royal Adelaide Hospital, Adelaide, South Australia, Australia; 3 Intensive Care Unit, Austin Medical Centre, Melbourne, Victoria, Australia; 4 Intensive Care Unit, Royal North Shore Hospital, Sydney, New South Wales, Australia

## Abstract

**Background:**

To compare two approaches to the statistical analysis of the relationship between the baseline incidence of adverse events and the effect of medical emergency teams (METs).

**Methods:**

Using data from a cluster randomized controlled trial (the MERIT study), we analysed the relationship between the baseline incidence of adverse events and its change from baseline to the MET activation phase using quadratic modelling techniques. We compared the findings with those obtained with conventional subgroup analysis.

**Results:**

Using linear and quadratic modelling techniques, we found that each unit increase in the baseline incidence of adverse events in MET hospitals was associated with a 0.59 unit subsequent reduction in adverse events (95%CI: 0.33 to 0.86) after MET implementation and activation. This applied to cardiac arrests (0.74; 95%CI: 0.52 to 0.95), unplanned ICU admissions (0.56; 95%CI: 0.26 to 0.85) and unexpected deaths (0.68; 95%CI: 0.45 to 0.90). Control hospitals showed a similar reduction only for cardiac arrests (0.95; 95%CI: 0.56 to 1.32). Comparison using conventional subgroup analysis, on the other hand, detected no significant difference between MET and control hospitals.

**Conclusions:**

Our study showed that, in the MERIT study, when there was dependence of treatment effect on baseline performance, an approach based on regression modelling helped illustrate the nature and magnitude of such dependence while sub-group analysis did not. The ability to assess the nature and magnitude of such dependence may have policy implications. Regression technique may thus prove useful in analysing data when there is a conditional treatment effect.

## Introduction

Following the landmark reports from the Institute of Medicine (IOM), numerous programs designed to improve patient safety have been introduced [1-6]. Rigorous evaluation of such programs provides a considerable challenge. Organisational theory recognizes that the effectiveness of health care interventions is likely to be system dependent. Therefore, understanding system specific organizational characteristics might be important in evaluating the effectiveness of interventions [7-10].

The medical emergency team (MET) was first introduced in Australia in the early 1990s. Its main aim is to reduce unexpected deaths, cardiac arrests and unanticipated Intensive Care Unit (ICU) admissions [11-14]. The MET and similar systems have now been widely adopted [15-17]. Single centre studies using historical controls have supported the effectiveness of the MET [18-26].

However, statistical analysis of data from the Medical Early Response & Intervention Therapy (MERIT) study, a 23-hospital cluster randomised controlled trial of the MET system, failed to show a difference in the aggregate incidence of unexpected cardiac arrests, unexpected deaths and unanticipated ICU admissions between MET and control hospitals [27]. The primary MERIT statistical analysis protocol was based on main effect analysis. Although it used the baseline incidence of adverse events as a covariate, it did not test for an interaction effect between treatment allocation and the baseline incidence of the study outcomes.

The baseline incidence of a specified outcome is an important hospital performance characteristic that may predict the magnitude of improvement in response to an intervention [28]. It varied greatly in MERIT. This raises questions regarding what might be the correct statistical approach to the analysis of the MERIT study results. Unfortunately, there is no established and widely accepted statistical approach that can be applied under these circumstances. Yet the choice of statistical method might well affect the interpretation of the study findings. In this setting, a comparison of statistical approaches might illustrate the impact of the choice of statistical technique on data interpretation and have useful implications for the analysis of similar studies in the future.

The aim of this study was to compare a regression based approach with subgroup analysis in terms of its results and empirical interpretation when the baseline outcome proved to be a continuous variable and treatment effect modifier. Accordingly, we developed a methodology that incorporated regression-modelling techniques and applied it to the MERIT study data. With this methodology, we studied the relationship between the baseline incidence of adverse events and the change that occurred during preparation for and activation of METs. We then compared the findings identified with this approach with those obtained from conventional subgroup analysis.

## Methods

The sample recruitment, size calculation, ethical approval, and randomisation scheme for the MERIT study have been described previously [27]. The primary outcome for the MERIT study was the aggregate incidence (adverse events divided by number of eligible patients admitted to the hospital during the study period) of the three adverse events: 1) cardiac arrests without a pre-existing not for resuscitation (NFR) order, 2) unplanned ICU admissions, and 3) unexpected deaths (deaths without an NFR order) occurring in general wards. Secondary outcomes were the incidences of each individual adverse event. Data collection was conducted during a two-month *baseline *period. This was followed by a four month standardised *implementation *period during which education was delivered on the concepts and practice changes required with the introduction of the MET and then by a six month study *activation *period during which the MET system was operational [27]. The MERIT study was approved by the Ethics Committee of the University of New South Wales.

Data collection was conducted in control hospitals during the same time periods. The conduct of the study was not publicised in the control hospitals, and the management and resuscitation committees of the control hospitals agreed that the operation of their cardiac arrest teams would continue unchanged during the study.

### Statistical Methods

To test our hypothesis, we used the previously published MERIT data. We set the change in incidence of adverse events as the dependent variable. We then tested for interaction effects (both linear and quadratic) between the baseline incidence of the primary and secondary outcomes and treatment allocation (MET versus control). We used an analytically weighted regression model. We weighted this model by the number of admissions during the study period. This weighting is an extension of the weighted-t test often used in cluster randomised controlled trials [29-31]. This initial approach established the existence of significant linear and quadratic interaction effects for the primary outcome, unexpected cardiac arrests and unplanned ICU admissions. Thus, we conducted the statistical analyses for control hospitals and MET hospitals, separately as described below.

### Statistical modelling given significant interaction effects

We analysed the primary outcome and secondary outcomes in the same way. First, we fitted a quadratic model. If the model detected a significant quadratic effect, these results were presented together with the results of the linear effect model for comparison. If the model detected no significant quadratic effect, a linear effect model only was fitted and presented. This was done to minimize the problem of multi-co linearity. This model showed that only unexpected cardiac arrests had negative linear slopes for both groups. We examined the statistical difference between these two slopes by testing the interaction effect between treatment group and the baseline incidence of unexpected cardiac arrests. We then addressed the issues of a) small sample size, b) lack of normality, and c) possible heteroscedasticity. We did this by comparing the analytically weighted regression modelling results using two different methods: the ordinary least square method and the heteroscedasticity-consistent covariance matrix estimation method (HC3) [31,32]. We also assessed the potential confounding effects of both teaching status and hospital location. We used a hybrid of forced entry and a blocked backward elimination multivariate regression model. The baseline incidence (linear or quadratic) was forced into the model. The block included teaching status and hospital location. The probability of the block exclusion from the final model was set at 0.15. We analytically weighted the regression model by admission volume. We present the results as the predicted regression curves with 95%CI bands, as well as the original data points for each hospital.

### Conventional subgroup analysis given significant interaction effects

For the purpose of this analysis, we followed convention and split the sample into two groups: those hospitals equal to or above the median baseline value and those hospitals below the median baseline value for a given outcome. We then used the weighted-t test for the analysis of both sub-groups separately.

All the analyses were performed using Stata™ 9.2 [33].

## Results

The baseline data for hospital and patient characteristics and the numbers for each event have been presented previously [27]. The baseline characteristics of the MET and control hospitals were similar.

Table 1 shows the results of the interaction effects between treatment (MET versus control) and the baseline incidence of the primary and secondary outcomes. There were significant quadratic interaction effects between treatment allocation and the baseline incidence of the primary outcome, unplanned ICU admissions and unexpected deaths. There was no significant interaction effect for unexpected cardiac arrests.

**Table 1:** Analytically weighted regression results testing the quadratic interaction effects between treatment and baseline incidence for primary outcome

	Change in incidence of primary outcome	P	Change in incidence of unexpected cardiac arrests	P	Change in incidence of unplanned ICU admissions	P	Change in incidence of unexpected deaths	P
MET versus control	-4.461	0.168	-2.630	0.090	-1.185	0.504	-1.637	0.004**
Baseline incidence of the outcome (per 1000 admissions)	-1.168	0.144	-2.345	0.053	-1.051	0.056	-2.076	<0.001**
Linear interaction effect between MET and baseline incidence of outcome	1.797	0.043*	2.114	0.088	0.964	0.147	1.658	0.006**
Baseline incidence of outcome squared	0.125	0.014*	0.262	0.226	0.063	0.090	0.256	0.036*
Quadratic interaction effect between MET and baseline incidence of outcome	-0.139	0.012*	-0.378	0.104	-0.097	0.039*	-0.322	0.021*
Constant	6.632	0.035*	3.201	0.039*	2.397	0.113	2.046	<0.001**
Observations	23		23		23		23	
R-squared	0.725		0.873		0.618		0.898	

* P < 0.05;

† Changes in incidence for primary outcome were calculated as events per 1000 admissions.

**Figure 1:**
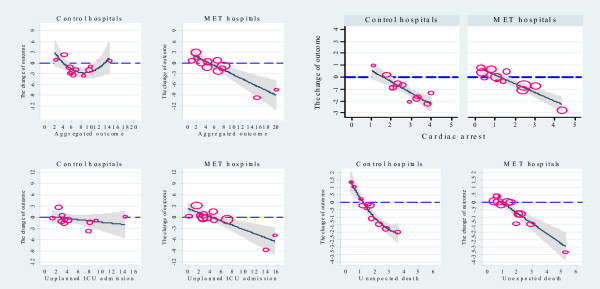
Relationship between baseline and change in incidence (y axis) for all outcomes during the study period*.* 95%CI band of the predicted curve as well as the original data points presented; the sizes of the scatter points in each graph were drawn proportional to the volume of admissions during the study period; baseline incidence and change in incidence are presented as events per 1000 admissions; for only primary outcome and unexpected death in control hospitals, the figures suggest a quadratic relationship.

Baseline, study and change in incidence from baseline to study period for the primary and secondary outcomes showed large variability (Table 2). The relationship between baseline incidence, and its change, for both primary and secondary outcomes is presented in Table 3. During the study period, hospital teaching status and location had no significant impact on this relationship (Table 4). Accordingly, we presented the results from the models with baseline incidence only and the predicted curves for these relationships are shown in Figure 1. In MET hospitals, 5 out of 8 Pearson correlation coefficients between baseline, implementation and study period for every outcome were greater than 0.83 with the lowest value at 0.65. In MET hospitals, the greater the baseline incidence of the primary outcome, the greater its reduction during study period. For every 10 additional baseline events per 1000 admissions, there was an additional reduction of 5.92 events (59.2%). Furthermore, the baseline incidence of the primary outcome accounted for 71% of the variance of this change. In comparison, in control hospitals, it accounted for 53% of variance. Sensitivity analysis showed that even after removing the two hospitals with the highest baseline incidence for the primary outcome, the findings were not qualitatively different.

**Table 2:** Incidence of primary and secondary outcomes in individual hospitals

	Primary outcome*			Cardiac arrests*			Unplanned ICU admissions*			Unexpected deaths*		
	
Control Hospitals	Baseline	Study	Change	Baseline	Study	Change	Baseline	Study	Change	Baseline	Study	Change
1	2.07	3.05	0.98	1.03	2.03	1.00	1.03	0.85	-0.19	0.52	1.53	1.01
2	3.91	6.32	2.41	1.74	1.93	0.19	2.17	4.80	2.63	1.45	1.31	-0.14
3	5.23	4.21	-1.02	2.29	1.75	-0.55	4.08	3.36	-0.72	1.15	0.94	-0.21
4	5.47	2.64	-2.83	2.02	1.15	-0.87	2.59	1.60	-1.00	2.74	0.95	-1.79
5	5.87	3.53	-2.34	3.25	1.47	-1.78	3.40	2.88	-0.52	1.70	0.60	-1.10
6	5.98	2.73	-3.24	3.76	1.54	-2.22	3.07	1.48	-1.59	2.22	0.74	-1.48
7	6.83	5.00	-1.82	3.32	1.69	-1.63	2.93	3.44	0.52	3.51	1.50	-2.02
8	8.42	5.00	-3.42	1.97	1.11	-0.85	7.58	3.89	-3.69	1.40	1.02	-0.39
9	9.70	7.82	-1.87	2.49	1.88	-0.61	7.90	6.62	-1.28	1.66	1.45	-0.21
10	9.99	9.17	-0.82	2.85	0.85	-2.00	9.04	8.15	-0.89	0.95	1.19	0.24
11	14.37	14.98	0.62	3.95	2.65	-1.30	14.37	14.45	0.09	0.36	1.72	1.36
MET Hospitals												
12	0.58	1.31	0.73	0.29	1.11	0.82	0.00	0.30	0.30	0.58	1.01	0.43
13	1.60	4.65	3.05	0.37	0.78	0.41	1.35	4.41	3.05	0.37	0.45	0.08
14	1.85	3.42	1.56	0.46	0.45	-0.02	1.85	2.38	0.52	0.46	0.89	0.43
15	2.95	3.22	0.27	1.03	1.04	0.02	2.57	2.45	-0.12	0.64	0.68	0.04
16	3.99	2.90	-1.09	2.35	1.24	-1.10	2.47	2.24	-0.23	0.82	0.66	-0.16
17	4.26	4.77	0.51	0.82	1.49	0.67	2.62	3.22	0.60	1.48	1.49	0.02
18	6.53	4.44	-2.09	4.35	1.62	-2.73	3.27	3.15	-0.12	2.18	1.38	-0.79
19	6.53	7.34	0.81	3.05	2.34	-0.70	4.35	5.81	1.46	2.03	1.27	-0.76
20	7.55	6.24	-1.31	1.56	2.05	0.49	4.43	3.68	-0.75	2.86	1.37	-1.50
21	7.89	7.16	-0.73	2.45	1.78	-0.67	6.98	6.40	-0.58	1.27	1.27	0.00
22	15.36	5.86	-9.51	1.40	1.07	-0.33	13.97	5.32	-8.64	1.86	0.40	-1.46
23	19.83	12.37	-7.46	1.04	0.75	-0.29	15.66	10.87	-4.78	5.22	1.88	-3.34

* Baseline incidence and change in incidence are presented as events per 1000 admissions.

**Table 3:** Weighted quadratic or linear regression models to predict changes in incidence of primary and secondary outcomes during the study period

	Change of primary outcome			Change of unexpected cardiac arrests		Change of unplanned ICU admission		Change of unexpected death		
	**Control: quadratic effect**	**Control: linear effect**	**MET^†^**	**Control**	**MET^†^**	**Control**	**MET^†^**	**Control: quadratic effect**	**Control: linear effect**	**MET^†^**
Baseline incidence (per 1000 admissions)	-2.168 (0.017)*	-0.135 (0.562)	-0.592 (0.001)**¶	-0.945 (<0.001)**	-0.736 (<0.001)**	-0.161 (0.313)	-0.556 (0.002)**	-2.076 (0.002)**	-1.039 (<0.001)**	-0.676 (<0.001)**
Baseline incidence squared	0.125 (0.020)*							0.256 (0.048)*		
Constant	6.632 (0.041)*	-0.518 (0.753)	2.789 (0.005)**	1.487 (0.009)**	0.932 (0.002)**	0.215 (0.804)	2.214 (0.007)**	2.046 (0.001)**	1.182 (0.002)**	0.587 (0.004)**
R-squared	0.532	0.039	0.710	0.782	0.851	0.112	0.643	0.923	0.870	0.819

Note: P values (in the parentheses) for the regression coefficients and the constant; control hospitals showed a quadratic effect for only the primary outcome and unexpected deaths.

* P < 0.05; ** P < 0.01;

¶The sensitivity analysis by removing the two hospitals with highest baseline incidence in MET hospitals produced a regression coefficient for baseline incidence as -0.416 with p = 0.034;

^†^Only the MET hospitals showed a linear effect for all of the four outcomes.

**Table 4:** Weighted quadratic or linear regression models to predict changes in incidence of primary and secondary outcomes during the study period after adjusting for teaching status and location of the hospitals

	Change of primary outcome			Change of unexpected cardiac arrests		Change of unplanned ICU admission		Change of unexpected death		
	**Control: quadratic effect**	**Control: linear effect**	**MET^†^**	**Control**	**MET^†^**	**Control**	**MET^†^**	**Control: quadratic effect**	**Control: linear effect**	**MET^†^**
Baseline incidence (per 1000 admissions)	-2.235 (0.042)*	-0.110 (0.692)	-0.523 (0.002)**	-0.980 (<0.001)**	-0.725 (<0.001)**	-0.085 (0.639)	-0.465 (0.010)**	-1.990 (0.021)*	-1.130 (<0.001)**	-0.680 (<0.001)**
Baseline incidence squared	0.128 (0.046)*							0.229 (0.216)		
Teaching vs. non-Teaching	0.521 (0.714)	0.338 (0.856)	-0.862 (0.568)	-0.130 (0.706)	-0.592 (0.212)	0.069 (0.960)	-0.285 (0.846)	-0.098 (0.816)	-0.460 (0.200)	-0.141 (0.695)
Rural vs. Urban	0.531 (0.792)	-0.761 (0.766)	-2.787 (0.129)	-0.739 (0.133)	-0.430 (0.393)	-2.051 (0.317)	-2.582 (0.158)	-0.107 (0.813)	-0.347 (0.437)	-0.338 (0.366)
Constant	5.411 (0.258)	-0.455 (0.928)	7.108 (0.106)	2.628 (0.034)*	2.497 (0.068)	1.998 (0.600)	5.231 (0.211)	2.290 (0.065)	2.557 (0.047)*	1.230 (0.242)
R-squared	11	11	12	11	12	11	12	11	11	12

Note: P values (in the parentheses) for the regression coefficients and the constant; control hospitals showed a quadratic effect for only the primary outcome.

* P < 0.05; ** P < 0.01;

^†^Only the MET hospitals showed a linear effect for all of the four outcomes.

For both MET and control hospitals, the greater the baseline incidence of unexpected cardiac arrests detected, the greater its reduction during the study period. For every 10 additional baseline cardiac arrests per 1000 admissions, there was an additional reduction of 9.45 and 7.36 arrests for control and MET hospitals, respectively. Furthermore, the baseline incidence of unexpected cardiac arrests accounted for 78.2% and 85.1% of the variance of this change, respectively. The interaction effect test showed no statistical difference. The baseline incidence had no impact on the change in unplanned ICU admissions in control hospitals. In contrast, in MET hospitals, there was a significant linear relationship between the baseline incidence and its change during the study period. In MET hospitals, for every 10 additional baseline events per 1000 admissions, there was an additional reduction of 5.56 events (55.6%). Furthermore, the baseline incidence of unplanned ICU admissions accounted for 64.3% of the variance of this change. In comparison, in control hospitals, it only accounted for 11.2% of this variance.

The baseline incidence showed a significant quadratic relationship with the change in unexpected deaths in control hospitals and a linear relationship in MET hospitals. In MET hospitals, for every 10 additional baseline unexpected deaths per 1000 admissions, there was an additional reduction of 6.76 events (67.6%). The baseline incidence accounted for 81.9% of the variance of this change. In comparison, in control hospitals, it accounted for 92.3% of this variance.

**Figure 2:**
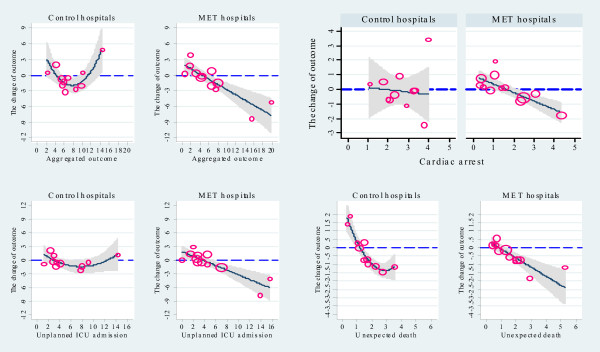
Relationship between and the baseline and changes in incidence (y axis) for all outcomes during the implementation period*. * 95%CI band of the predicted curve as well as the original data points presented; the sizes of the scatter points in each graph were drawn proportional to the volume of admissions during the implementation period; baseline and change in incidence were presented as events per 1000 admissions; for only primary outcome and unexpected death in control hospitals, the figures suggest a quadratic relationship.

The relationship between baseline incidence and its change between baseline and implementation periods for each outcome is shown in Table 5. These show that, in MET hospitals, for all outcomes, the greater the baseline incidence, the greater the reduction. In contrast, control hospitals showed a quadratic trend for primary outcome and unexpected deaths. These relationships are shown in Figure 2.

**Table 5:** Weighted quadratic or linear regression models to predict the changes in incidence of primary and secondary outcomes during the implementation period

	Change of primary outcome			Change of unexpected cardiac arrests		Change of unplanned ICU admission			Change of unexpected death		
	**Control: quadratic effect**	**Control: linear effect**	**MET^†^**	**Control**	**MET^†^**	**Control: quadratic effect**	**Control: linear effect**	**MET^†^**	**Control: quadratic effect**	**Control: linear effect**	**MET^†^**
**Baseline incidence (per 1000 admissions)**	-2.393 (0.008)**	0.099 (0.699)	-0.497 (0.001)**	-0.163 (0.761)	-0.585 (<0.001)**	-0.941 (0.046)*	-0.081 (0.547)	-0.504 (<0.001)**	-2.810 (0.001)**	-0.844 (0.003)**	-0.587 (<0.001)**
**Baseline incidence squared**	0.154 (0.006)**					0.061 (0.056)			0.487 (0.006)**		
**Constant**	7.342 (0.022)*	-1.379 (0.455)	2.191 (0.008)**	0.292 (0.841)	0.952 (0.004)**	2.160 (0.089)	0.053 (0.943)	1.767 (0.007)**	2.700 (0.001)**	1.067 (0.026)*	0.497 (0.019)*
**R-squared**	0.645	0.017	0.717	0.011	0.731	0.411	0.042	0.719	0.870	0.653	0.732

Note: P values (in the parentheses) for the regression coefficients and the constant; control hospitals showed a quadratic effect for only the primary outcome and unexpected deaths,.

* P < 0.05; ** P < 0.01

^†^Only the MET hospitals showed a linear effect for all of the four outcomes.

### Conventional subgroup analyses results

There was no significant interaction effect (p = 0.081) for a dichotomized modifier using the baseline median value as cut-off. Logically, therefore, there was no need to conduct further subgroup analysis. Nevertheless, the following subgroup analysis was presented for demonstrative purpose. Table 6 shows the results of statistical analysis using the weighted-t test for sub-groups after splitting them according to median baseline values. None of the outcomes showed any statistical significance between MET and control hospitals for both subgroups, except for a borderline significant effect for unexpected cardiac arrests in the subgroup with an incidence lower than the median baseline incidence.

**Table 6:** Subgroup analysis results using medians of the baseline incidences as the cut-off values

	Change in incidence of primary outcome	Change in incidence of unexpected cardiac arrests	Change in incidence of unplanned ICU admissions	Change in incidence of unexpected deaths
**Group with baseline incidence ≥ medians**				

**Weighted difference (MET versus Control)**	0.402	0.536	0.091	0.222
P	(0.776)	(0.315)	(0.937)	(0.636)
Number of hospitals	12	12	12	12
**Group with baseline incidence < medians**				
**Weighted difference (MET versus Control)**	1.597	0.726	1.258	-0.118
P	0.200	0.042*	0.325	0.678
Number of hospitals	11	11	11	11

* Significant at 5%

## Discussion

We applied a statistical approach that incorporates regression-modelling techniques to the analysis of data from the MERIT study; a cluster randomized controlled trial of the implementation of Medical Emergency Teams (METs). We compared this approach to one based on the conventional method of using a median cut off value to separate groups and then performing subgroup analysis. We found that an approach incorporating regression modelling detected a significant effect of the baseline incidence of the adverse events upon the subsequent effect of introducing METs. In contrast, conventional subgroup analysis did not. The findings based on regression modelling suggest that the baseline incidence of cardiac arrests, unplanned ICU admissions, and unexpected deaths has a significant association with their subsequent reduction after the introduction of a MET. They also indicate that the magnitude of this reduction was proportional to their baseline incidence. Finally, they demonstrate that the choice of statistical analysis plan can significantly affect the interpretation of the outcome data obtained during MERIT [34]

The MERIT study paper was designed to compare the incidence of the primary and secondary outcomes during the study period and powered according to the expected size of the therapeutic effect and the expected incidence and variance of the primary outcome [35]. Accordingly, all the analyses were designed and conducted based on a main effect model with adjustment for baseline incidences. This is similar to an ANCOVA approach [36]. Thus, the baseline incidences of both primary and secondary outcomes were adjusted for in the model, but only as covariates. No interaction effects were tested for. Such strategy didn't examine whether the treatment effect was influenced by the baseline incidence of the study outcomes. It didn't examine the possibility that the outcomes would be, to some extent, affected by their baseline incidence of adverse events (baseline hospital performance). It is plausible for many interventions where the relative risk reduction may relate to different baseline incidences of the primary outcome. In MERIT trial, we could address this issue by empirically testing for the interaction effect between treatment allocation and the baseline incidence of study outcomes. We tested for both linear and quadratic interaction effects in order to avoid potential misspecification of true interactions. Misspecification refers to situations when significant interaction effects and/or higher order effects are omitted from a model. In this case, a simplified model with only a main effect and/or linear effect is a simple but possibly inaccurate reflection of the relationship under investigation. The rationale for introducing a higher order interaction effect was that there was an insufficient theoretical basis to believe that any interaction effect should be linear. This is analogous to introducing a quadratic effect for continuous variables in a main effect model.

Given the above considerations, we set out to test whether choice of statistical technique affected our ability to detect the impact of baseline incidence of adverse events on the effect of introducing MET systems. We used regression modelling techniques to explore the relationship between the baseline incidence of adverse events and its change. The main advantage of using the change of the outcome as the endpoint is in its intuitive interpretation. That is, a lot of system and policy initiatives aim to affect change. The changes incurred by the intervention are often the outcomes we want to understand. Also given that the distribution of baseline incidences was balanced and the before-after correlation was high for the primary endpoint, the efficacy gain of using an ANCOVA approach may have been negligible [36]. We assessed MET and control hospitals separately after we found significant interaction effects and found that, for the primary outcome, unplanned ICU admissions and unexpected deaths, the baseline incidence and their subsequent changes were related in a different way in MET hospitals compared to control hospitals. In MET hospitals, the relationship between the baseline incidence and its change was linear. In control hospitals, this relationship was quadratic for the primary outcome and for unexpected deaths. It was absent for unplanned ICU admissions. We also found that, for MET hospitals, these observations started to apply during the education and training period well before the MET system had been activated. A similar education effect on outcome after introduction of a MET system has been previously suggested [19].

The MERIT main-effect analysis, an unconditional valid analysis, showed that benefit of MET may be small on average. Our conditionally analysis showed that the benefit may still be large among those hospitals with high baseline incidences of the study outcomes. The observations derived from statistical modelling suggest a 'proportionality effect'. That is, the relative change in the incidence of an outcome was similar across all levels of baseline incidence, but the absolute change became greater as baseline incidence increased. This phenomenon has been described with other interventions [28]. For example, a recent study of the impact of quality of care interventions in 3000 U.S. hospitals found that, for 16 out of the 17 process-of-care measures, hospitals with a low level of performance at baseline had the greatest improvement [28].

Our results are consistent with the notion that the effectiveness of interventions in complex organizations may be dependent on specific local characteristics. They also suggest that baseline characteristics of individual hospitals should be explored when assessing the possible effectiveness of interventions directed at system change. The strong predictive effect and the magnitude of the variance of improvement explained by the baseline incidence of outcome variables also suggest that such outcomes may be useful indicators of quality of care. They are relatively easy to measure, define, and report upon in a timely fashion.

The regression towards the mean effect may explain the baseline effect seen in MET hospitals with our statistical modelling approach. Regression toward the mean effect, sometimes called the regression effect, is a statistical principle concerning the relationship between two linked measurements, *x *and *y*. It states that if *x *is above its mean, then the associated *y *is likely to be closer to its mean than *x *was. The conventional way to assess data for the presence of this effect in MERIT would be to compare the pattern of results in MET hospitals with those in control hospitals [37]. The difference in the pattern shown in Figure 1 &2 between MET and control hospitals suggests that the distribution of outcomes is unlikely to be explained by such an effect. In addition, the range of regression coefficients for all outcomes increased in magnitude during the study period. Furthermore, in MET hospitals, the correlations between baseline, implementation and study period were high. Such higher correlation makes it less likely that the results are due to regression towards the mean [38]. Moreover, as we adopted a stratified (by teaching status and geographic location: urban versus metropolitan) and blocked randomisation scheme, the baseline incidences distributions were balanced between MET and control hospitals [27]. Stratified randomization and baseline balance also make it less likely that a regression towards the mean effect explains our findings.

Our statistical approach avoided the possibility of misspecification by incorporating a higher order interaction effect. Secondly, by not splitting the sample using a median value cut-off, our approach decreased the problems associated with loss of statistical power. It may be possible to effectively carry out a sufficiently powered conventional subgroup analysis in a very large randomized controlled trial. However, the MERIT study had only a 25% power to detect a 30% difference in the primary outcome and more than 100 hospitals would have been needed to give it sufficient power. These features of the MERIT study make it unlikely that one could detect a statistical difference when comparing 5 to 6 hospitals within two arms. A further aspect of our modelling approach is that, although we analysed MET and control hospitals separately, our conclusions are based on the comparison of the same results between two groups. This approach compared the relationship of baseline performance with study outcome between control and treatment groups and used all data to draw conclusions. Subgroup analysis, instead, assessed treatment effects in both lower and higher baseline performance groups, respectively. This aspect is a further critical factor in making it less likely that the different patterns seen were due to the regression-toward-the-mean phenomenon. The advantage of this regression approach over subgroup analysis may be that it allows for smooth linear or nonlinear relationships with baseline variables. These smooth relationships may be plausible in some settings such as in the MERIT study. However, one potential disadvantage of our approach is that, due to the existence of a significant quadratic interaction effect, it may not be feasible to provide a unique treatment effect estimate (such as absolute risk difference) as would be the case with a conventional approach. Furthermore, the existence of a non-linear interaction effects means that the incremental value of the treatment effect is dependent on the value of the baseline incidence. The baseline period was short compared to the implementation and study periods. This may have influenced our findings. However, we found a strong association between the baseline incidence and its change. The strength of this association provides evidence that the baseline incidence had sufficient predictive validity.

Our analysis was performed post hoc. Furthermore, the number of hospitals included in the study was relatively small. However, sensitivity analysis showed that even after removing the two hospitals with the highest baseline incidence for the primary outcome, the findings were not qualitatively different. Nonetheless, our results should be considered preliminary and they should be confirmed in future studies with similar design.

## Conclusions

In summary, we used regression modelling to test the hypothesis that the baseline incidence of cardiac arrests, unplanned ICU admissions, and unexpected deaths has a significant influence on the change in their incidence that could be achieved through the introduction of a MET. Our findings support this hypothesis. We also found that the magnitude of this reduction was proportional to the baseline incidence of adverse events such that the higher the baseline incidence, the greater the absolute reduction. These differences were consistent with the initial findings of the significant interaction effects. We found that these observations were in contrast to those obtained using conventional sub-group analysis. They suggest that the choice of statistical analysis can significantly affect the interpretation of the findings of the MERIT study. They also raise concerns about the robustness of conventional sub-group analysis in similar settings.

## List of abbreviations

MET: Medical Emergency Team. ICU: Intensive Care Unit;MERIT: Medical Early Response Intervention & Therapy; ANCOVA: Analysis of Covariance.

## Competing interests

The authors declare that they have no competing interests.

## Authors' contributions

JC contributed to the conceptualisation, all data analyses and prepared the first draft of the paper. AF, RB, KH and SF contributed to the conceptualisation and critical writing of the paper. All authors approved the final draft of the paper and have the access to the data used in generating the paper. The draft was approved by the ANZICS Clinical Trials Group Executive Committee.
